# Botanical Volatiles Selection in Mediating Electrophysiological Responses and Reproductive Behaviors for the Fall Webworm Moth *Hyphantria cunea*

**DOI:** 10.3389/fphys.2020.00486

**Published:** 2020-05-29

**Authors:** Peng-Hua Bai, Hong-Min Wang, Bao-Sheng Liu, Min Li, Bai-Ming Liu, Xi-Shu Gu, Rui Tang

**Affiliations:** ^1^Institute of Plant Protection, Tianjin Academy of Agricultural Sciences, Tianjin, China; ^2^State Key Laboratory for Biology of Plant Diseases and Insect Pests, Institute of Plant Protection, Chinese Academy of Agricultural Sciences, Beijing, China; ^3^College of Economics and Management, Shanxi Agricultural University, Taigu, China; ^4^Tianjin Key Laboratory of Animal and Plant Resistance, Tianjin Normal University, Tianjin, China; ^5^State Key Laboratory of Integrated Management of Pest Insects and Rodents, Institute of Zoology, Chinese Academy of Sciences, Beijing, China

**Keywords:** *Hyphantria cunea*, host volatiles, GC-MS, SEM, electroantennogram, reproductive behavior

## Abstract

Host-plant volatiles play vital roles for insects to locate foraging, mating, and oviposition sites in the environment. As one of the devastating invasive forestry pests, *Hyphantria cunea* causes a great annual loss in China, and understanding its chemical ecology is an important task. The current research was done in terms of chemical analysis, electrophysiology, and behavioral assays on *H. cunea* to assess its olfactory reception toward host-plant volatiles. A screen of possible common host volatiles was done, targeting on five favored hosts of *H. cunea*, harvesting six potential bioactive compounds from a total of 78 odorant components. Six types of antennal sensilla were investigated on their distributions on the antennae, and sexual dimorphism was described. *H. cunea* showed responses to all selected host-related volatiles in electroantennogram tests, and linalyl butyrate elicited the strongest responses. Furthermore, mating rates in adult pairs that are exposed to dibutyl phthalate and phytol have been significantly increased, while oviposition rates and female fecundity were not influenced. The results of the current study provide initial evidence showing that universal host-derived volatile cues are essential for *H. cunea* moth in terms of mating, which can also provide insights into the development of botanical attractants.

## Introduction

In recent years, there have been extensive studies on the role of plant-derived compounds that regulate interactions between herbivorous insects and their host plants (Joseph and Carlson, 2015; Bisch-Knaden et al., 2018; Turlings and Erb, 2018; Guo and Wang, 2019). Several studies have shown that plant volatiles play a significant role in mating and host recognition in phytophagous insects by influencing antennal sensitivities to chemical components (Xu et al., 2001; Reed and Landolt, 2002; Natale et al., 2004; Bleeker et al., 2011; Gundappa et al., 2016). Various works have indicated that host-derived volatiles were successfully deployed in the management of agricultural and forestry pests by the development of botanical attractants, push–pull strategies, and modulation of reproductive behaviors. The botanical attractants based on volatiles from host plants are known to effectively attract both sexes of herbivorous insect pests, which plays an important role in monitoring and control of insect pests including *Bactrocera dorsalis*, *Helicoverpa armigera*, *Cydia pomonella*, and *Frankliniella occidentalis* (Light et al., 2001; Li et al., 2005; Siderhurst and Jang, 2006; Singh and Mullick, 2010; Abdullah et al., 2015; Gregg et al., 2016). The push–pull strategies with botanical volatiles have also been widely utilized in pest control, for instance, *Delia radicum* in cabbage, *Diaphorina citri* Kuwayama in citrus, and Aphididae species in wheat-pea strip (Kergunteuil et al., 2015; Yan et al., 2015; Xu et al., 2018). Furthermore, several studies have shown that the chemical composition of host plant plays an integral part in mating and oviposition behaviors in various insects, including Lepidoptera, Hemiptera, and Coleoptera (Shelly, 2001; Paschen et al., 2012; Juma et al., 2016).

The fall webworm *Hyphantria cunea* (Durry) (Lepidoptera: Arctiidae) is a devastating invasive insect that is native to North America. *H. cunea* was first recorded in Liaoning Province in China in 1979 and later it has widely expanded its distribution to Beijing, Tianjin, Hebei, Shandong, Shanxi, Jiangsu, and Anhui provinces (Yang et al., 2006; Chen et al., 2014; Zhang et al., 2016a). It has been reported that the occurrence of *H. cunea* in China reached a 20.9% annual increase, and this pest has been causing massive damage to forests, orchards, and even crops because of its polyphagous trait (Yang et al., 2006; Su et al., 2008; Zhang et al., 2016b). Currently, more than 400 plant species are recorded to serve as hosts for *H. cunea* worldwide (Schowalter and Ring, 2017). Several methods including biopesticides, parasitoid wasps, and sex pheromone traps have been used to monitor and control *H. cunea* (Su et al., 2008; Yang et al., 2008; Kim et al., 2011; Saruhan et al., 2014). Specifically, due to its TYPE II sex pheromone structures, synthetic pheromone luring of *H. cunea* was applied at a higher cost than other lepidopteran pests (Hill et al., 1982; Su et al., 2008; Schowalter and Ring, 2017). Sex pheromone monitoring of *H. cunea* in China nowadays has been solely dependent on imported luring products, which raised the demands of developing botanical attractants/synergists or related push–pull strategy for large-scale IPM needs (Bai et al., 2017).

There has been less previous evidence regarding the influences of plant volatiles toward *H. cunea.* Some volatile compounds from host plant (*Morus alba*, *Malus spectabilis*) and area-dependent non-host plant (*Robinta pseudoacacia*) have been identified (Tang et al., 2012; Bai et al., 2018a, b). Given the fact that this polyphagous pest may infest different plant hosts in its habitats depending on local vegetation distributions (Yang et al., 2010), screen for universal host cues can help to explore the chemical ecology of this moth. On the other hand, recent research has reported morphology of its antennal sensilla, but information on the distribution of sensillar types has not yet been tackled (Zhang et al., 2019). To date, a number of questions on olfaction of *H. cunea*, such as its key host volatiles as well as the electrophysiological sensitivity on antennae and how plant volatiles affect its reproductive behaviors, remain to be answered. In the current work, we identified 78 host volatile compounds from five favored host species of *H. cunea* in China and narrowed down to six key components for later tests. The distributions of six sensilla types on antennae of *H. cunea* were investigated by scanning electron microscopy (SEM). Later, the electroantennogram (EAG) test against selected host-plant odorants has been used to investigate the sensitive volatiles that have potential effects on the behaviors. To test the reproductive behavioral responses of *H. cunea* to volatile compounds associated with host plants, we studied the effects of sensitive volatiles on the mating and oviposition of *H. cunea*. The results from the current research can provide insights into a better understanding of the olfactory perception of *H. cunea*. Moreover, selection of the effective botanical components can be used for future utilizations as a botanical attractant and pheromone synergetics in ecological-based control of this devastating pest in the fields.

## Materials and Methods

### Insects and Plants

*H. cunea* were obtained from the Chinese Academy of Forestry, where a laboratory population of *H. cunea* using wild moths was established from Qinghuangdao, Hebei Province, China. The pupae were stored in a climate chamber at 25 ± 1°C and R.H. 50–60% under a 16L:8D photoperiod until emergence. Newly emerged moths were immediately separated by gender and kept at a density of five adults per 500-ml beaker with 5% sugar solution provided. One- to three-day-old virgin moths were fasted for 3–6 h before being used in electrophysiological and behavioral tests.

Plant leaves from *M. alba*, *Fraxinus chinensis*, *Populus alba*, *Ailanthus altissima*, and *M. spectabilis* were collected in forestry experimental sites of the Tianjin Institute of Plant Protection in Wuqing, Tianjin (39°25′33.3″N, 116°57′35.7″E). Leaf samples were immediately brought back to the lab where extraction process was carried out within 1 day.

### Chemical Analysis

Tested leaf samples were cut into fine pieces and boiled with water with 1:1 w/w for 4–6 h to prepare the water distillations, followed by extraction with hexane. Upper phases of the extracts were then collected and desiccated with anhydrous sodium sulfate drying agent columns. Prepared essential extracts were analyzed at 1 ml/min through an Agilent Technologies 5973MD mass spectrometer coupled with an Agilent Technologies 6890N gas chromatography system (Santa Clara, CA, United States) equipped with a quartz capillary column (HP-5MS, 30 m × 0.25 mm × 0.25 μm; J&W Scientific, Palo Alto, CA, United States). Volatile traces were identified by crosschecking with the mass spectrum fragment database (NIST 2.0) with GC/MSD ChemStation (Agilent) and selected components for later tests were confirmed against standard chemical spectrum patterns.

### Scanning Electron Microscopy

The antennae from the head capsule of emerged adults (*n* = 12; sex ratio, 1:1) were placed in 2% glutaraldehyde solution fixed overnight at 4°C. After three washes at room temperature with 0.1 M PBS, antennae were incubated for 2 h at 4°C and then washed three times with 0.1 M PBS. The specimens were dehydrated through a series of graded ethanol (30, 50, 70, 80, and 90%), followed by three washings in 100% ethanol. After drying in a critical point drier (Bal-Tel CPD 030), antennae were mounted on aluminum stubs, taking care to place them with different orientations, to obtain views of the ventral and dorsal aspects and of both of the lateral sides. Then, the mounted specimens were sprayed with gold (Bal-Tel SCD 005) and observed with SEM (FEI Quanta 200). The sensilla were classified according to their morphology, shape, and size, following Schneider (1964) and Zacharuk (1980).

### Chemicals

The 11 *M. alba*-related volatiles were previously identified (Tang et al., 2012), and they were selected in this study as references for the later chemicals; detailed information can be found in Supplementary Table S1. The reason we put these 11 chemicals in the comparison was that they were from the favorite plant of *H. cunea* and they compose a full spectrum of bioactive volatiles of this host species toward the pest. We suggested that the amplitudes elicited by these chemicals may provide a baseline response for investigation on the newly tested six volatiles in this work. The six additional components (phytol, n-pentatriacontane, linalyl butyrate, palmitic acid, α-linolenic acid, and dibutyl phthalate, all 95% minimum purity) used in this study were selected by either referring to the GC-MS results or to the studies on host plant *M. spectabilis* and area-dependent non-host plant *R. pseudoacacia* of *H. cunea* (Bai et al., 2018a, b). All of these six synthetic standard components were obtained from Meryer Chemical Technology Co., Ltd. (Shanghai, China). For EAG experiment, the components (phytol, linalyl butyrate, α-linolenic acid, dibutyl phthalate, and other 11 *M. alba* volatiles) were dissolved in paraffin oil for dosage response trials (10 μl applied to a 0.8 × 2 cm filter paper with concentrations at 0.001, 0.01, 0.1, 1, 10 μg/μl, respectively), and n-pentatriacontane and palmitic acid were dissolved at the same dosages in hexane as they were insoluble in paraffin oil. The other 11 reference volatiles were prepared at four concentrations of 0.1–100 μg/μl (equal to 1–1000 μg in dosages). The dosages of the six components for mating and oviposition experiments were decided according to the results of the EAG experiment.

### Electrophysiology

The EAG method was adopted to test the receptivity of the antenna of male and female moths to the volatile compounds. Each antenna was carefully excised at both extremes and immediately placed within the indifferent electrodes with Spectra 360 conductive gel. The signals were passed through an IDAC-2 data acquisition controller unit and recorded by a computer using a software package (Syntech, Kirchzarten, Germany). The stimulus was delivered into a purified air stream (30 ml/s) by a gas control unit (Syntech CS-55). The gas outlet faced the antenna with 1 cm distance from it. For each sample, 10 μl was applied to a 0.8 × 2 cm filter paper in a Pasteur pipette. Antenna from both sexes of *H. cunea* were stimulated for 0.8 s with an interval time of 30 s. The same antenna was used to test all of the concentrations of a single compound. Each concentration was tested with six different antennae per gender. The sequence of the tested compound was provided starting from the weakest concentration and followed by increasing concentrations. The control was added before and after stimulation by each concentration of the tested compounds. Recorded EAG data were fed into IDAC-2 and analyzed with EAG 2000 software (Syntech).

### Reproductive Behavioral Response

To evaluate the reproductive behavioral responses of moths to plant-derived volatiles, single-pair courtship assays were used. We observed the effect of the electrophysiologically active compounds (at the concentration that elicited the strongest EAG response) on mating and oviposition of *H. cunea*. Green rubber septa (The West Company, Phoenixville, PA, United States) were loaded with 100 μl of the tested volatile mixtures at the selected concentration. Septa containing 100 μl of solvent and a blank septum were used as controls. A pair (one male, one female) of 1- to 3-day-old virgin moths was placed in a beaker (500 ml, *d* = 9.4 cm, *h* = 12.4 cm) together with one septum at 25 ± 1°C, R.H. 50–60%, and 16L:8D. The number of pairs that mated within 24 h was observed and counted. Mating rate was defined as the proportion of the mated pairs in 10–15 pairs tested independently, and oviposition rate indicates proportion of mated females that performed oviposition behaviors. Finally, egg numbers were counted after 48 h in oviposition female pairs as female fecundity. A total of four replicates were conducted for each treatment.

### Statistical Analysis

The EAG data were standardized to include background influences of solvents before means ± SEM were calculated for parametric tests. Specifically, the relative EAGs to a test stimulus (Sr) were calculated as Sr = 2 Sc/(R′ + R″), where Sc is the absolute amplitude of the stimuli and R′ and R″ are the mean responses to the reference substances before and after stimulation (Hou and Yan, 1995; Zhu et al., 2017). The same data of each chemical at 10 μg were used in both baseline comparison and dosage comparison. Means ± SEM were calculated for measurement data (sensilla parameters) and proportion data (behavioral tests) before testing in a parametric model. Counts data for sensilla were tested with chi-square tests. Statistics for means were analyzed with either two-tailed *t*-test (two treatments) or two-tailed GLM (>2 treatments) followed by *Dunnett* against controls or *Tukey* HSD at *P* = 0.05. SPSS version 17.0 (IBM, Chicago, IL, United States) software was used for statistical analysis.

## Results

### Common Volatiles in Five Favored Host Plant

**FIGURE 1:**
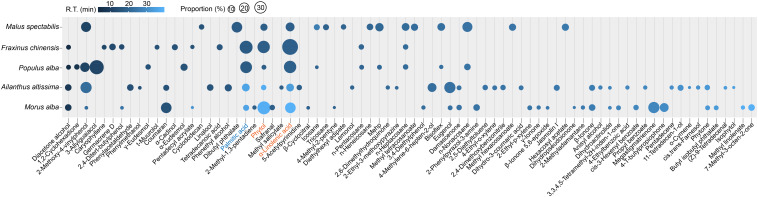
Bubble chart showing overall traces of chemical extracts from five major host plants of *H. cunea* by GC-MS analysis. The chemical blends were extracted by hexane from water-distillated foliage samples. Bubble size indicates proportion (%) of each component in the blend from each host. Bubble color indicates retention time (R.T. min) of each corresponding component. Phytol and α-linolenic acid are highlighted in orange as they are observed in all five host blends. Palmitic acid is highlighted in blue as it is included in four of the five host blends with a high proportion at 12.32% ± 4.9 (mean ± SEM). Original source data for the figure is provided in Supplementary Data Sheet S2A.

A total 78 components were identified through GC-MS approach in the extracts of host plants for *H. cunea* (Figure 1). Most of the components existed in only one to two blends, but phytol and α-linolenic acid were observed in the GC-MS traces of all five blends from *M. alba*, *F. chinensis*, *P. alba*, *A. altissima*, and *M. spectabilis*. α-Linolenic acid occupied the highest proportion of 17.73 ± 5.6% (mean ± SEM) in the volatiles, and phytol was the second highest at 10.6 ± 4.9%. Palmitic acid existed in four of five plant volatile blends including *M. alba*, *F. chinensis*, *P. alba*, and *A. altissima*, occupying overall 9.8 ± 4.5% of the blends. 2-Methoxy-4-vinylphenol was also observed in four plants of *M. alba*, *P. alba*, *A. altissima*, and *M. spectabilis* with covering 7.73 ± 3.16% of the volatiles. Diacetone alcohol was also found in *M. alba*, *F. chinensis*, *P. alba*, and *A. altissima* with a proportion of 1.96 ± 0.80%. Based on these results, we suggested that the top three components including phytol, α-linolenic acid, and palmitic acid may have vital roles in host communications of *H. cunea* as they were the most common and abundant volatiles in the tested favored host plants. We then decided to include these three chemicals in the successive tests. The main reason for selecting the other n-pentatriacontane, linalyl butyrate, and dibutyl phthalate for testing was based on our previous works which revealed their potentially high electrophysiological activities toward *H. cunea* (Bai et al., 2018a, b). We sought to find whether some behavioral/ecological significance of these six chemicals can be otherwise exhibited in the later trials.

### Sensillar Types and Distribution

The antenna of *H. cunea* moth is composed of the scape, pedicel, and flagellum; the flagellum of the female is composed of 34–42 sub-segments (mean ± SEM = 40 ± 0.95) and that of the male is 35–42 (38.6 ± 1.24) (Supplementary Figures S1A–D). The estimated length of the flagellum was 5467 ± 247.0 μm for males and 5644.3 ± 151.3 μm for females. No difference was observed between genders in terms of length of flagellum (Supplementary Figure S1E) (*t*-test, *t*_10_ = 0.61, *P* = 0.55). The SEM investigations of the antenna revealed six types of sensilla, including sensilla chaetica, sensilla trichodea, sensilla basiconica, sensilla coeloconica, sensilla squamiformia, and sensilla böhm bristles (Supplementary Figure S2). The antennal morphology and types of sensilla as well as identifiers in *H. cunea* are identical with recently reported works on the same species (Zhang et al., 2019).

**FIGURE 2:**
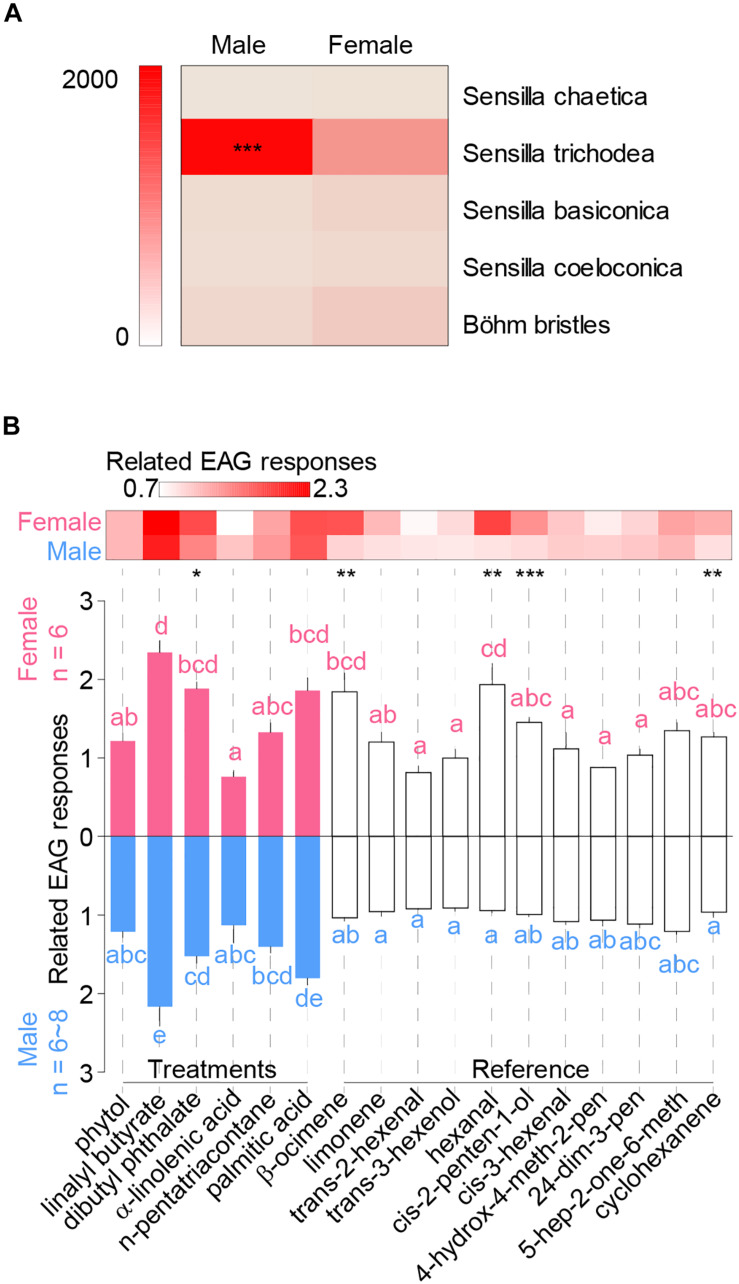
Distribution of antennal sensilla and EAG responses of *H. cunea*. **(A)** Heatmap indicates overall numbers per antenna of each sensillar type in either male or female antennae. **(B)** Comparisons of *H. cunea* EAG responses among volatile components at the concentration of 1 μg/μl. Treatment chemicals included phytol, linalyl butyrate, dibutyl phthalate, α-linolenic acid, n-pentatriacontane, and palmitic acid. The other 11 chemicals were host volatiles that were used as references against treatments. Heatmap blocks indicate means of standardized EAG responses. Asterisks indicate significant differences between genders. Lowercase letters indicate significant differences among tested chemicals to female or male antennae. Error bars indicate + SEM.

Distributions of sensilla types were not even between genders, and males bear significantly more sensilla trichodea per antenna than females (Figure 2A) (chi-square test, χ_4_ = 405, *P* < 0.0001), indicating its potential role in sex pheromone olfactory reception. Males had significant longer sensilla trichodea (*t*-test, *t*_10_ = 2.57, ^∗^*P* = 0.028) and shorter sensilla chaetica than female adults (Supplementary Figure S2N) (*t*-test, *t*_10_ = 2.8, ^∗^*P* = 0.018). Basal width of sensillar types were the same between genders expected for sensilla chaetica (Supplementary Figure S2N) (*t*-test, *t*_10_ = 2.9, ^∗^*P* = 0.016).

### Electrophysiological Responses of *H. cunea* to Host Volatiles

All chemicals were compared at the concentration of 1 μg/μl, and gender bias for each chemical was shown in the heatmap (Figure 2B). Female moths showed significant higher responses than males when tested with dibutyl phthalate, β-ocimene, hexanal, cis-2-penten-1-ol, and cyclohexanene (Figure 2B) (GLM, dibutyl phthalate: *t* = 2.95, *P* = 0.014; β-ocimene: *t* = 3.78, *P* = 0.0026; hexanal: *t* = 4.30, *P* = 0.001; cis-2-penten-1-ol: *t* = 6.6, *P* < 0.0001; cyclohexanene: *t* = 3.22, *P* = 0.0073). While no male bias response was observed among all tested chemicals. Within the six new chemicals, linalyl butyrate, dibutyl phthalate, and palmitic acid elicited significantly higher EAG responses in both genders of adults when compared with most other chemicals (Figure 2B) [GLM and Tukey HSD, females: *F*_(__16,85__)_ = 10.8, *P* < 0.0001; males: *F*_(__16,107__)_ = 14.5, *P* < 0.0001].

**FIGURE 3:**
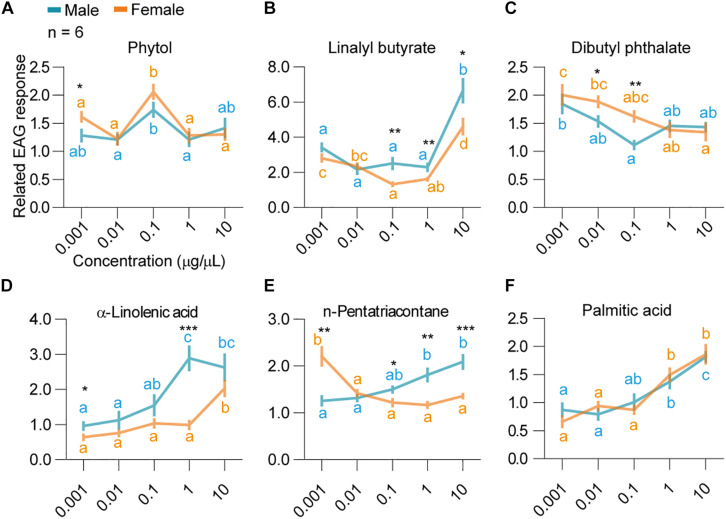
Dosage responses of *H. cunea* adults EAG to selected host volatile components. Concentrations included 0.001, 0.01, 0.1, 1, and 10 μg/μl. **(A)** Adult EAG responses to phytol. Different lowercase letters indicate significant differences among tested dosages in both genders. Asterisk indicates significant difference between genders at 0.001 μg/μl concentration. **(B)** Adult EAG responses to linalyl butyrate. Differences in females and males were shown in different letters. Differences between genders at 0.1, 1, and 10 μg/μl were shown as asterisks. **(C)** Adult EAG responses to dibutyl phthalate. Differences in females and males were shown in different letters. Differences between genders at 0.01 and 0.1 μg/μl were shown as asterisks. **(D)** Adult EAG responses to α-linolenic acid. Differences in females and males were shown in different letters. Differences between genders at 0.001 and 1 μg/μl were shown as asterisks. **(E)** Adult EAG responses to n-pentatriacontane. Differences in females and males were shown in different letters. Differences between genders at 0.001, 0.1, 1, and 10 μg/μl were shown as asterisks. **(F)** Adult EAG responses to palmitic acid. Differences in females and males were shown in different letters. All error bars indicate ± SEM.

In the later dosage response tests, significant differences in the EAG responses were observed among the doses for all components (Figures 3A–F and Supplementary Figure S3). Strong and significant positive correlation was observed between EAG responses and dosages in males against α-linolenic acid in males [Linear regression, *r*^2^ = 0.84, *F*_(__4,22__)_ = 15.99, *P* = 0.028], n-pentatriacontane [Linear regression, *r*^2^ = 0.94, *F*_(__4,22__)_ = 53.6, *P* = 0.0053], and palmitic acid [Linear regression, *r*^2^ = 0.85, *F*_(__4,22__)_ = 17.38, *P* = 0.0251] (Figures 3D–F). Female EAG responses positively correlated with dosages when tested with palmitic acid (Linear regression, *r*^2^ = 0.89, *F*_4,22_ = 23.51, *P* = 0.0167) (Figure 3F). EAG responses of dibutyl phthalate [*F*_(__4,25__)_ = 5.6, *P* = 0.002] and n-pentatriacontane in females [*F*_(__4,25__)_ = 17.9, *P* < 0.0001] were decreased along with increase of dosages (Figures 3C,E). The highest responses for phytol were at the dosage of 0.1 μg/μl, and a decrease was observed afterward (Figure 3A) [GLM and Tukey HSD, female: *F*_(__4,25__)_ = 11.6, *P* < 0.0001; male: *F*_(__4,25__)_ = 3.79, *P* = 0.0153].

When comparing the standardized EAG responses of both sexes of *H. cunea* to all six volatile compounds at the same concentration, male bias was observed for linalyl butyrate at 0.1–10 μg/μl, and α-linolenic acid at 0.001 and 1 μg/μl (Figures 3B,D). In contrast, female bias was observed for phytol at 0.001 μg/μl and dibutyl phthalate at 0.01–0.1 μg/μl (Figures 3A,C). For n-pentatriacontane, female bias was observed at 0.001 μg/μl, while male bias was observed during 0.1, 1, and 10 μg/μl (Figure 3E). Last, female and male moths did not show any significant sex-specific difference in their EAG responses to palmitic acid [two-way ANOVA, *F*_(__4,50__)_ = 1.157, *P* = 0.93].

### Mating Rates Are Increased by the Volatiles

**FIGURE 4:**
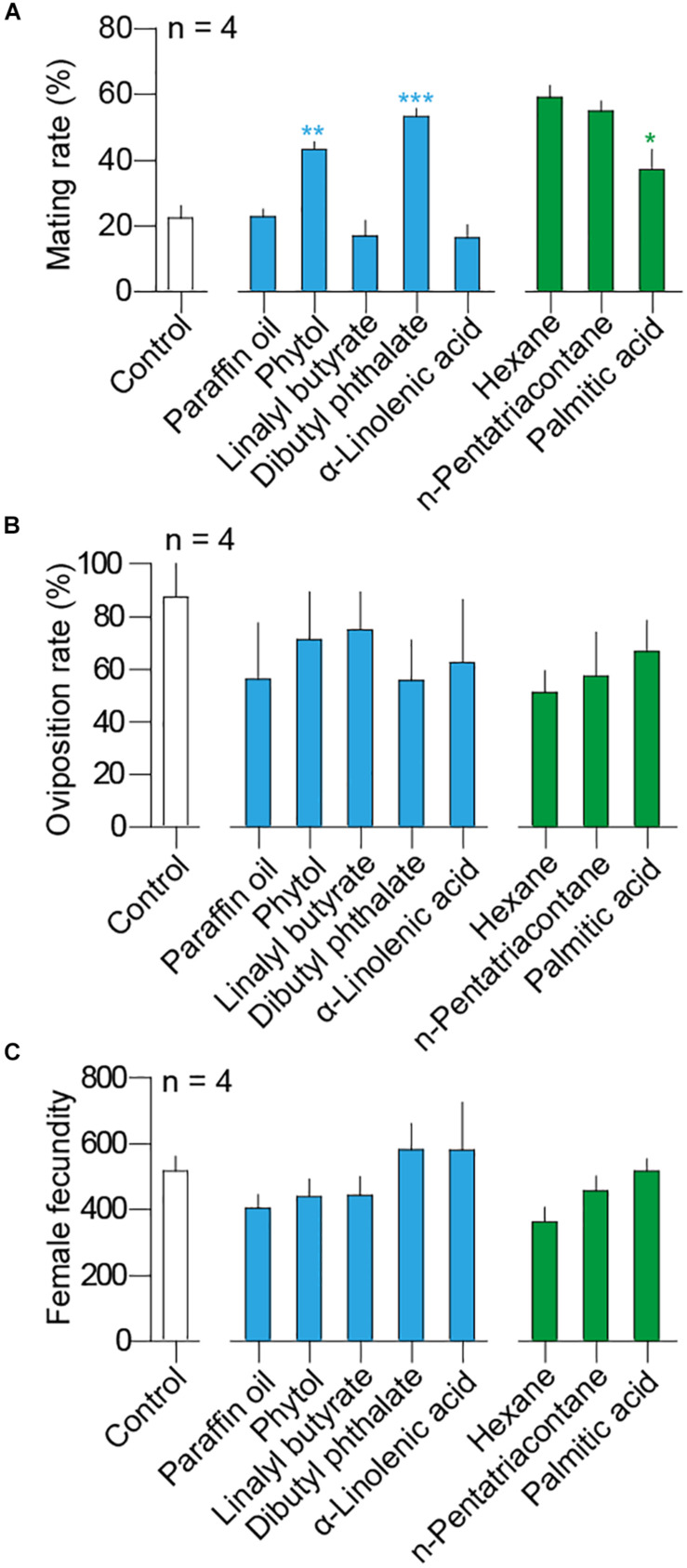
Influences of selected host volatile components to *H. cunea* adults in **(A)** mating rates, **(B)** oviposition rates, and **(C)** female fecundity of *H. cunea*. Either paraffin oil (blue) or hexane (green) was used as solvent. White bars indicate blank control, which was used to assess background behavioral rates. Significant differences between treatment and paraffin oil were indicated with blue asterisks. Significant differences between treatment and hexane was indicated with green asterisk. No significant difference was observed in terms of either oviposition rates or female fecundity among tested treatments. All error bars indicate + SEM.

In the reproductive behavioral assays, background mating rate was 22.5 ± 3.69% (mean ± SEM) in blank control. Solvent as paraffin oil had elicited mating rates at 22.86 ± 2.19% and hexane was at 59.07 ± 3.60% (Figure 4A). Compared to solvent, moth adults were more inclined to mate after they have been exposed to host-plant volatiles including dibutyl phthalate (*t-*test, *t*_6_ = 9.48, *P* < 0.0001) and phytol (*t-*test, *t*_6_ = 6.33, *P* = 0.0007), but no difference was observed between n-pentatriacontane and its solvent hexane (*t-*test, *t*_6_ = 0.88, *P* = 0.41) (Figure 4A). The mating rates of moths exposed to dibutyl phthalate and phytol have reached 55.83 ± 15.11% and 71.25 ± 18.07%, respectively, at 24 h, more than twofold than the control and the paraffin oil solvent. On the other hand, palmitic acid decreased mating rates of adults compared with its solvent hexane (*t-*test, *t*_6_ = 3.05, *P* = 0.0226) (Figure 4A).

Over 80% of the mated females conducted oviposition afterward when no chemical was applied (Figure 4B). However, all volatiles including two solvents did not influence the oviposition rates of *H. cunea* females [GLM and Dunnett test, *F*_(__8,27__)_ = 0.49, *P* = 0.85] (Figure 4B). Background fecundity of *H. cunea* was 516.6 ± 45.87 eggs (mean ± SEM) per female in blank control treatment. When comparing the total number of eggs produced in different pairs, no significant difference was observed between the pairs exposed to volatiles and the control or the solvent [GLM and Dunnett test, *F*_(__8,27__)_ = 1.57, *P* = 0.15] (Figure 4C).

## Discussion

Plant volatiles serve as essential cues to insects searching for resources, mates, and oviposition sites (Juma et al., 2016; Hanks et al., 2018; Houjun et al., 2018; Xu and Turlings, 2018). The reception of host-plant volatiles in Lepidoptera is accomplished through a highly sensitive chemosensory system on the antenna, which is able to recognize and discriminate many different volatile chemicals (Gökçe et al., 2005; GöKçe et al., 2018; Molnár et al., 2017). The present electron microscopy study demonstrated that both sexes of *H. cunea* contain six different types of sensilla on their antenna, including sensilla trichodea, sensilla chaetica, sensilla basiconica, sensilla coeloconica, sensilla squamiformia, and sensilla böhm bristles, which revealed identical observations with previous studies (Zhang et al., 2019). Compared with other Lepidoptera, the morphology of the sensilla on the antenna of *H. cunea* is similar to related species, such as *H. armigera*, *Phauda flammans* Walker, *Dogwood Borer*, *Holcocerus hippophaecolus* Hua, and *Tuta absoluta* (Frank et al., 2010; Wang et al., 2015; Chang et al., 2016; Bawin et al., 2017; Liu et al., 2018). Antennal sensilla sexual dimorphism can be reflected by different length and width diameters of corresponding sensillar types (Zhang et al., 2019). Furthermore, we have also observed that distribution of sensillar types may vary between genders, which is intimately connected to feeding, mate location, oviposition, and other functions, indicating an especially ubiquitous form of intraspecific variation in moths (Allen et al., 2011). The function of sensilla has been reported to be primarily sensory, responsible for olfactory detection and perception of tactile signals. This involved various behaviors including habitat searching, host recognition, host location, host acceptance, copulation, and oviposition (Ansebo et al., 2005; Meng et al., 2012; Zuo et al., 2015). The sensilla trichodea are the most conspicuous and the most numerically abundant type of sensilla on the antenna of *H. cunea*. It is frequently shown that sex pheromone components and plant volatiles are usually detected by sensilla trichodea (Kaissling, 2004; Meng et al., 2012; Chang et al., 2016; Liu et al., 2018a, b), suggesting that sensilla trichodea may be responsible for distinguishing pheromone components and plant volatiles. There is also ample evidence that sensilla basiconica participate in recognition for plant volatiles (Lopes et al., 2002; Yang et al., 2012). The information of the literature on the function of sensilla is instructional significant for peripheral coding of plant-associated compounds for *H. cunea*.

Electrophysiological recordings have been applied to the study of peripheral coding of host-plant volatiles in *H. cunea* (Tang et al., 2012, 2016). Both sexes of *H. cunea* moths showed high sensitivity to the compounds tested that are potentially involved in host–moth interactions. These results are relatively consistent with a preliminary study; volatiles and their dosages elicited electro-physiological responses in the antenna of male and female moths (Tang et al., 2012, 2016). On the other hand, responses to olfactory cues in behavioral assays are not always consistent with EAG tests (Choo et al., 2018; Xiu et al., 2019). Our mating tests also revealed that linalyl butyrate and α-linolenic acid did not influence mating rates in *H. cunea*, although they exhibited significant EAG amplitudes. While we cannot exclude the possible role of these two chemicals in terms of feeding or host location, we still need to be cautious when talking about bioactive compounds without substantial evidence from behavioral tests. Furthermore, significant differences in the EAG responses were observed among the doses, which is consistent with other studies (Lu et al., 2017; Oseiowusu et al., 2020). For instance, the highest EAG responses were found with linalyl butyrate, α-linolenic acid, and palmitic acid at the highest dose; in contrast, the compounds dibutyl phthalate and n-pentatriacontane elicited a more robust response at lower doses. This differential odor sensitivity could be attributed to the presence of specific receptors that respond to particular categories of chemicals (Shields and Hildebrand, 2001). The higher EAG responses were found with linalyl butyrate than all other compounds in both sexes of the moths. Plant-associated ester compounds are more efficient for other moths, such as pear ester in *C. pomonella*, *Rhagoletis*, and *Dysaphis plantaginea* (Light et al., 2001; Linn et al., 2003; Van Tol et al., 2009).

The results obtained in our electrophysiological experiment prompted us to investigate the function of the compounds derived from plants on the reproduction system. In single-pair courtship assays, we found that the mating rate of *H. cunea* exposed to host-derived volatiles dibutyl phthalate and phytol were more than twice that from the control and paraffin oil. This is consistent with what has been found in the previous study regarding the effects of β-ocimene (host volatile) on mating rates of *H. cunea* moth pairs (Tang et al., 2016). These results suggested that host-derived volatile cues could promote mating behaviors in *H. cunea* moths. The same is true for females of *Plutella xylostella*, which rely heavily on host-plant volatiles for mating (Wee, 2016). Similarly, females of certain tephritid fruit fly species (Diptera: Tephritidae) show increased receptivity to mating after exposure to host fruit volatiles (Gerofotis et al., 2013; Bachmann et al., 2015). One of the most important factors is that females could emit more sex pheromone while exposing to plant volatiles, such as in cabbage looper moth *Trichoplusia* (Landolt et al., 1994), the corn earworm moth *Helicoverpa zea* (Raina et al., 1992), and the tobacco budworm *Heliothis virescens* (Raina et al., 1997). Also, the plant-provided signals deliver spatial information for insects to find mates. For example, plant volatiles are likely to serve as attractants to insects over a larger spatial range and pheromones are more likely to help guide the insects over a relatively short distance to mate in parasitoids (Xu and Turlings, 2018). Host-derived volatiles play an important role as oviposition stimulant for Lepidopterans, such as tobacco hornworm *Manduca sexta*, *Ostrinia latipennis*, *C. pomonella*, and the stem borer *Busseola fusca* (Mechaber et al., 2002; Li and Ishikawa, 2006; Lombarkia and Derridj, 2008; Juma et al., 2016). However, our results showed that female oviposition rates and fecundity of *H. cunea* did not show sensitivity toward the volatiles from host plants. In all, potential ecological-based trapping method can be developed by utilizing these common volatiles from host plants of *H. cunea*, and an olfactory peripheral coding mechanism and the molecular basis of this pest are worth looking into by future studies.

## Data Availability Statement

All datasets generated for this study are included in the article/Supplementary Material.

## Author Contributions

P-HB and RT designed the study. P-HB, H-MW, and ML conducted the SEM tests. P-HB, H-MW, and RT conducted the electroantennogram experiments. B-SL, B-ML, and X-SG conducted the indoor bioassays. RT, P-HB, and H-MW analyzed the data and drafted the manuscript with contributions from all authors.

## Conflict of Interest

The authors declare that the research was conducted in the absence of any commercial or financial relationships that could be construed as a potential conflict of interest.
