# Supplementary material for: Botanical Volatiles Selection in Mediating Electrophysiological Responses and Reproductive Behaviors for the Fall Webworm Moth Hyphantria cunea
Source: Front Physiol. 2020 May 29;11:486. doi: 10.3389/fphys.2020.00486 (PMC7273966; doi:10.3389/fphys.2020.00486)
Supplement: Supplementary file 1 [file Data_Sheet_1.docx]

Supplementary Materials for:

**Botanical Volatiles Selection in Mediating Electrophysiological Responses and Reproductive Behaviors for the Fall Webworm Moth *Hyphantria cunea***

Table S1

Figure S1-3

**Table S1. Chemicals used in the experiments**

| **Chemicals** | **Purity%** | **Source*** |
| --- | --- | --- |
| Paraffin oil | Analytically pure** | a |
| β-ocimene | 95% | b |
| Limonene | 95% | c |
| trans-2-hexenal | 99% | d |
| trans-3-hexe-1-ol | 97% | e |
| Hexanal | 95% | b |
| cis-2-penten-1-ol | 95% | c |
| cis-3-hexenal | 95% | b |
| 4-hydroxy-4-methyl-2-pentanone | 99+% | f |
| 2,4-dimethyl-3-pentanol | 99% | c |
| 6-methyl-5-hepten-2-one | 98% | c |
| Cyclohexanene | 99.80% | d |

*a: Beijing Chemical Factory, Beijing, PRC; b: Sigma-Aldrich, St. Louis, MO, USA; c: Tokyo Chemical Industry Co., Tokyo, Japan; d: J&K Chemica, Shanghai, PRC; e: Alfa Aesar, Ward Hill, MA, USA; f: Acros Organics, New Jersey, USA.

** tested with GC.


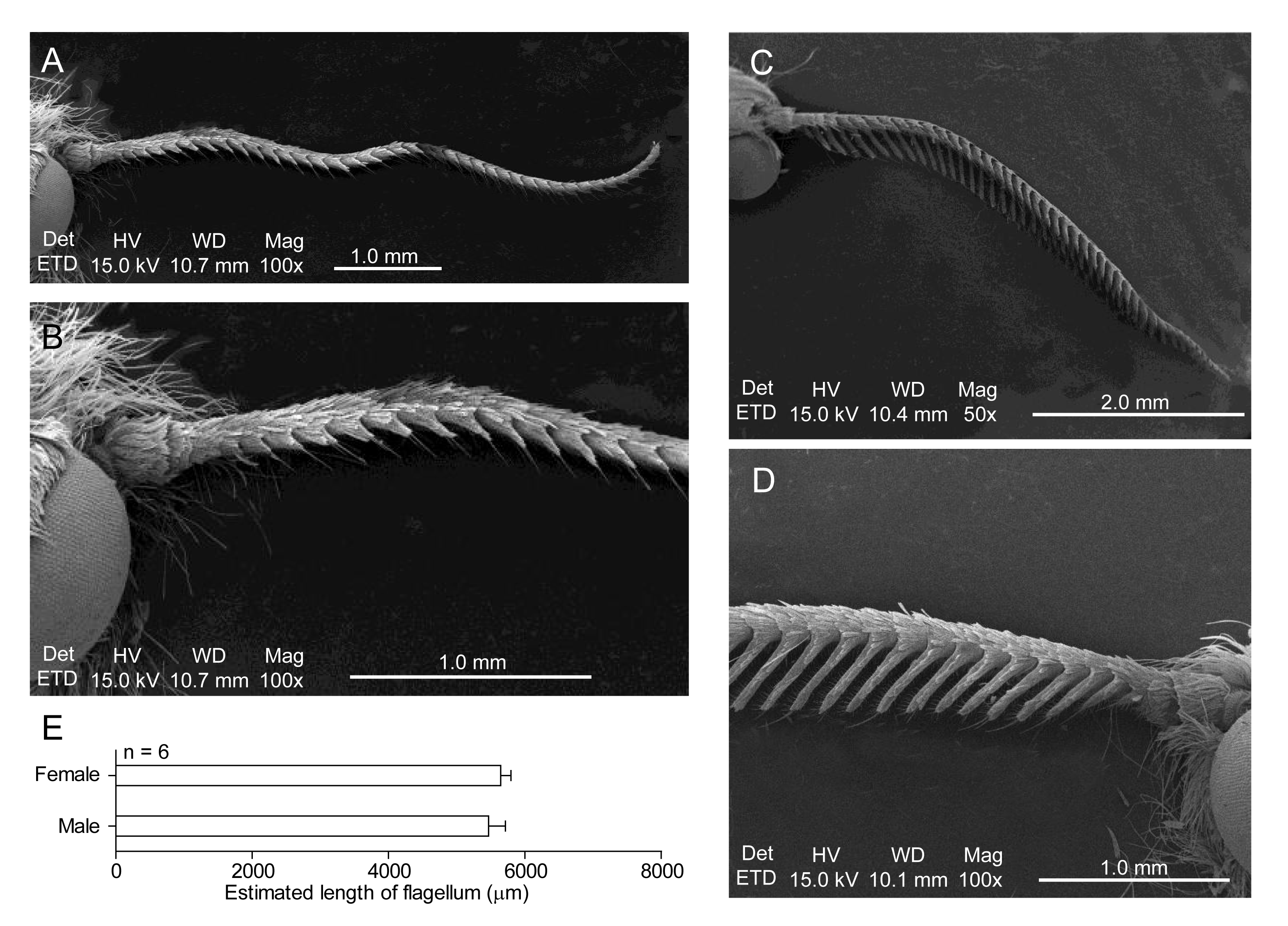


**Figure S1.** The antenna of male and female *H. cunea* adults. **(A**-**B)** Female adults. **(C**-**D)** Male adults. (E) Comparison of length of antennal flagellum between male and female adults. No significant difference was observed (*t* test, *t*_10_ = 0.61, *P* = 0.55). Error bars indicate + s.e.m.


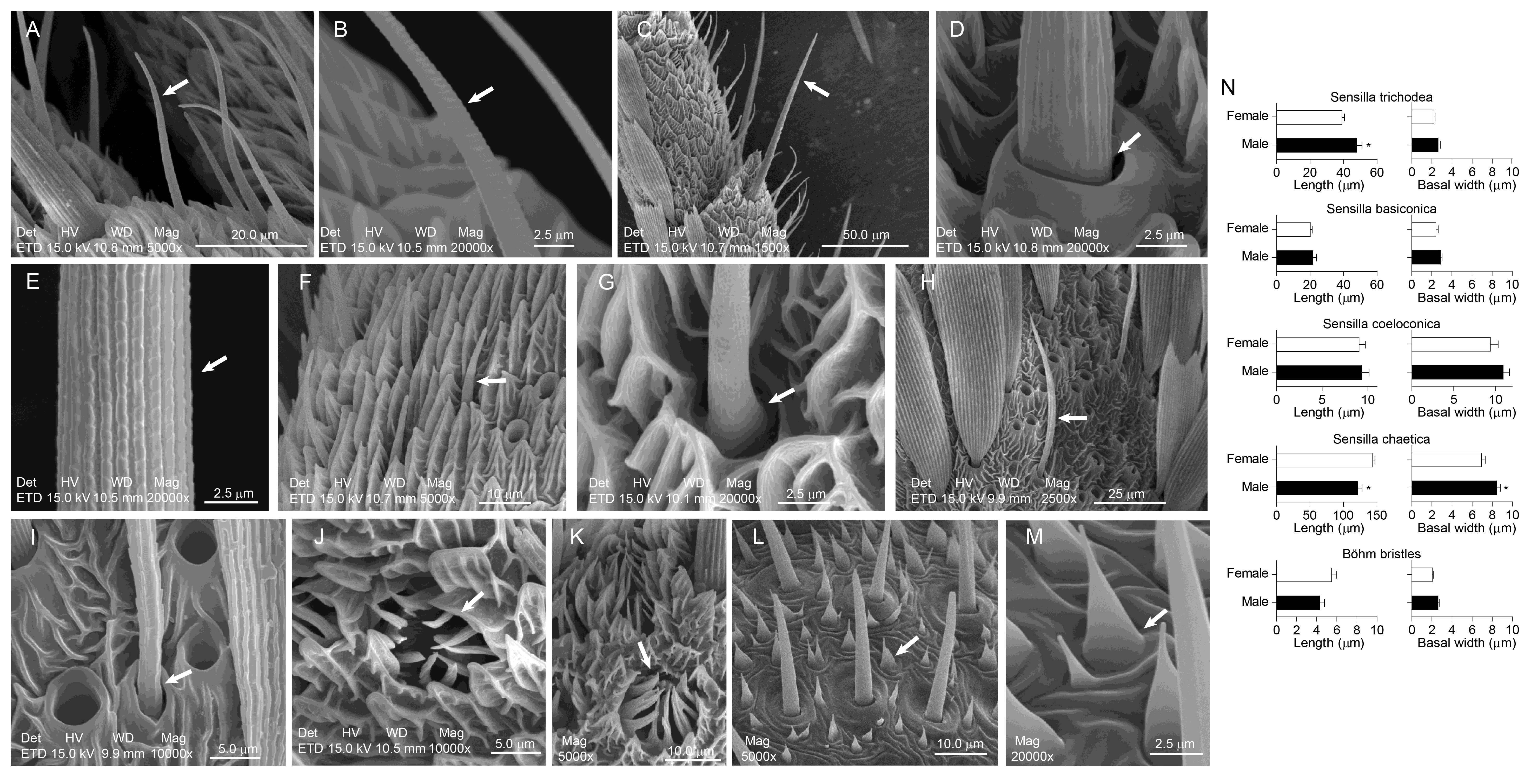


**Figure S2.** Representative SEM images of the sensilla on the antenna of *H. cunea* adults. **(A-B)** Sensilla trichodea; **(C-E)** Sensilla chaetica; **(F-G)** Sensilla basiconica; **(H-I)** Sensilla squamiformia; **(J-K)** Sensilla coeloconica; **(L-M)** Böhm bristles. **(N)** Morphological statistics of each type of sensilla in either male or female adults of *H. cunea*. Asterisks indicate significant differences between genders (*t* test, *: *P* < 0.05). Error bars indicate + s.e.m.


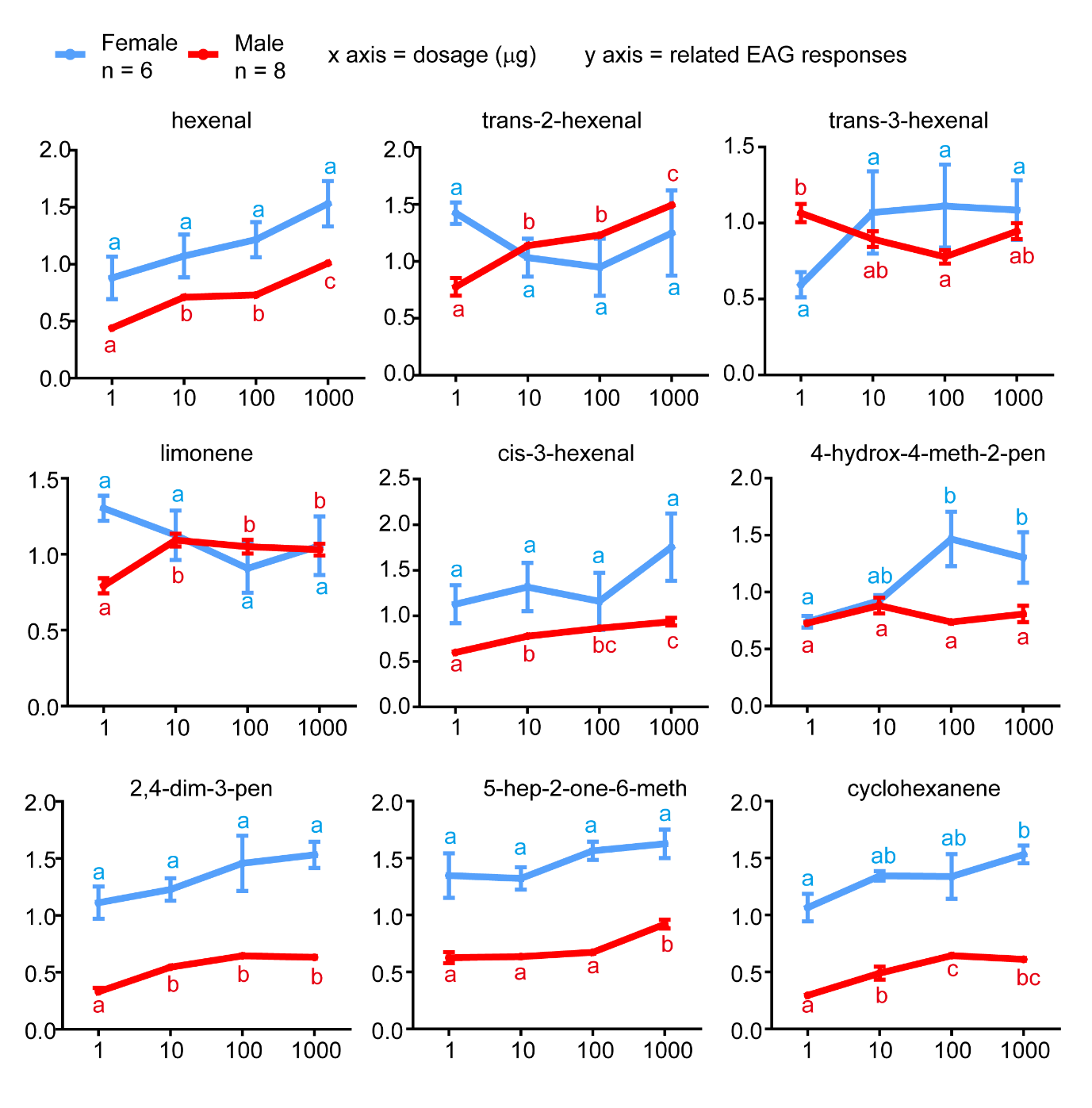


**Figure S3. Dosage responses of selected host volatile components to *H. cunea* adults in EAG tests.** Related responses were calculated according to the standard formula mentioned in method section. Lower case letters indicate significant differences among dosages at *P* = 0.05. Error bars indicate + s.e.m.
